# Advanced Glycation End Products (AGE) and Soluble Forms of AGE Receptor: Emerging Role as Mortality Risk Factors in CKD

**DOI:** 10.3390/biomedicines8120638

**Published:** 2020-12-21

**Authors:** Elena Dozio, Simone Vettoretti, Lara Caldiroli, Silvia Nerini-Molteni, Lorenza Tacchini, Federico Ambrogi, Piergiorgio Messa, Massimiliano M. Corsi Romanelli

**Affiliations:** 1 Department of Biomedical Science for Health, Laboratory of Clinical Pathology, Università degli Studi di Milano, 20133 Milan, Italy; lorenza.tacchini@unimi.it (L.T.); mmcorsi@unimi.it (M.M.C.R.); 2 Unit of Nephrology, Dialysis and Kidney Transplantation, Fondazione IRCCS Ca’ Granda Ospedale Maggiore Policlinico di Milano, 20122 Milan, Italy; simone.vettoretti@policlinico.mi.it (S.V.); lara.caldiroli@policlinico.mi.it (L.C.); piergiorgio.messa@unimi.it (P.M.); 3 Department of Clinical Microbiology, ASST Grande Ospedale Metropolitano Niguarda, 20162 Milan, Italy; silvia.nerini@unimi.it; 4 Department of Clinical Science and Community Health, Laboratory of Medical Statistics and Biometry “Giulio A. Maccacaro” Università degli Studi di Milano, 20133 Milan, Italy; federico.ambrogi@unimi.it; 5 Department of Clinical Science and Community Health, Università degli Studi di Milano, 20122 Milan, Italy; 6 Service of Laboratory Medicine1-Clinical Pathology, IRCCS Policlinico San Donato, San Donato Milanese, 20097 Milan, Italy

**Keywords:** advanced glycation end-products (AGE), chronic kidney disease (CKD), mortality, soluble receptor for AGE (sRAGE)

## Abstract

Advanced glycation end-products (AGE) can promote chronic kidney disease (CKD) progression and CKD-related morbidities. The soluble receptor for AGE (sRAGE) is a potential biomarker of inflammation and oxidative stress. Here, we explored the role of AGE, glycated albumin, sRAGE and its different forms, cRAGE and esRAGE, as prognostic factors for mortality in 111 advanced CKD patients. The median follow-up time was 39 months. AGE were quantified by fluorescence, sRAGE and its forms by ELISA. Malnutrition was screened by the Malnutrition Inflammation Score (MIS). The Cox proportional hazards regression model was used to assess the association of variables with all-cause mortality. Mean levels of sRAGE, esRAGE and cRAGE were 2318 ± 1224, 649 ± 454 and 1669 ± 901 pg/mL. The mean value of cRAGE/esRAGE was 2.82 ± 0.96. AGE were 3026 ± 766 AU and MIS 6.0 ± 4.7. eGFR correlated negatively with AGE, sRAGE, esRAGE and cRAGE, but not with cRAGE/esRAGE. Twenty-eight patients died. No difference was observed between diabetic and non-diabetic patients. Starting dialysis was not associated with enhanced risk of death. AGE, esRAGE and cRAGE/esRAGE were independently associated with all-cause mortality. AGE, esRAGE and cRAGE/esRAGE may help to stratify overall mortality risk. Implementing the clinical evaluation of CKD patients by quantifying these biomarkers can help to improve patient outcomes.

## 1. Introduction

Patients with chronic kidney disease (CKD) are at a higher risk of accelerated biological aging, malnutrition, cardiometabolic, musculoskeletal and cerebral complications, subsequent functional and quality-of-life impairments, and mortality [1,2,3,4].

In CKD patients, excessive oxidative stress, uremia, and chronic inflammation may increase the production of advanced glycation end-products (AGE), which in turn promote CKD-related morbidities and mortality. The damaging effects of AGE may be due in part to the direct modification and loss of function of those substrates that are involved in AGE formation, such as matrix proteins, in part to the activation of a pro-inflammatory response following the activation of the membrane-bound AGE receptor (RAGE) [5,6]. The accumulation of AGE participates to renal filtration alteration and glomerulopathy [5,7]. Animal studies supported the involvement of AGE in nephropathy, showing a thickening of the basement membrane and expansion of the mesangial layer after AGE injection [8]. The underlying molecular mechanisms following RAGE activation by AGE involve the production of pro-inflammatory cytokines and pro-oxidant molecules, extracellular matrix accumulation and glomerular hypertrophy [5,6,9]. The kidney normally clears circulating AGE, but these products accumulate both in diabetic and not diabetic nephropathy [10,11]. Furthermore, higher concentrations of AGE may contribute to renal function deterioration and increase cardiovascular risk and mortality in end-stage CKD and kidney transplanted patients [10,12,13].

Much attention has recently been paid to the soluble receptor for AGE (sRAGE) as a biomarker of inflammation, oxidative stress, atherosclerosis, heart failure and risk of kidney disease [14,15,16]. sRAGE is a decoy receptor that prevents AGE binding to membrane-bound RAGE and RAGE-related detrimental effects [17,18]. sRAGE is a circulating pool composed by two different forms: the cleaved RAGE (cRAGE), which derives from proteolytic cleavage of the membrane-bound RAGE (cRAGE), and the endogenous secretory RAGE (esRAGE), a splice variant with the extracellular domains but without the intracytoplasmic and transmembrane domains [19]. When AGE bind to RAGE, they increase RAGE expression, promote inflammation, downregulate the production of esRAGE and upregulate the levels of inflammatory enzymes, such as metalloproteases (MMP), that cleave the membrane-bound RAGE and increase cRAGE levels [20,21]. In addition, sRAGE accumulate in CKD due to the increased production and reduced kidney filtration. 

Recently, we reported an association between higher levels of sRAGE and mortality in hemodialysis and peritoneal dialysis patients [15]. Likely, glycated albumin (GA), an early precursor of AGE that is associated with the overall prognosis of patients in dialysis [22], may influence the prognosis of patients with advanced CKD. 

Whether AGE, sRAGE and its forms may be considered prognostic factors for mortality also in patients with advanced CKD not yet on dialysis is currently unknown, therefore the exploration of these associations will be the aim of the present work. Furthermore, to evaluate more comprehensively the impact of AGE in CKD patients, we will also evaluate the association of GA with all-cause mortality.

## 2. Experimental Section

### 2.1. Patients and Study Design 

We cross-sectionally evaluated 111 prevalent patients that were attending our outpatient clinic for advanced CKD between 9/2016 and 3/2018. Median follow-up time was 39 months [95% confidence interval (CI): 38 to 41]. Patients were selected according to the following criteria: age ≥ 65 years, CKD stages 3b to 5 [8 ≤ Glomerular filtration rate (eGFR) ≤ 45 mL/min/m^2^] not yet on renal replacement therapy and relatively stable eGFR over the previous 6 months (with less than 2 mL/min/1.73/m^2^ of variation). eGFR was estimated according to the CKD-EPI formula. We applied some exclusion criteria: active and advanced cancer, decompensated chronic liver diseases (advanced cirrhosis and/or ascites), severe heart failure (NYHA class III–IV), nephrotic syndrome, hypothyroidism or hyperthyroidism, malabsorption diseases and inability to cooperate. We also excluded all patients that were under treatment with immunosuppressive drugs or had a recent hospitalization (in the previous three months). Twenty-four hours urinary collection was started in the morning of the day preceding the visit. Biochemical parameters were sampled after an overnight fasting. The study was conducted according to the ICP Good Clinical Practices Guidelines and to the declaration of Helsinki, as revised in 2013, and it was approved by the Local Ethics Committee (N. 347/2010). All patients signed an informed consent.

### 2.2. Malnutrition Inflammation Score

Patients were screened for malnutrition by using the malnutrition inflammation score (MIS), as a validated scoring system for the assessment of malnutrition and inflammation in CKD patients [23]. A score of 4–7 is indicative of mild malnutrition and ≥8 of severe malnourishment [24].

### 2.3. sRAGE, esRAGE and cRAGE Quantification

sRAGE was measured by a commercial ELISA kit from R&D Systems (DY1145, Minneapolis, MN, USA). esRAGE was quantified by the ELISA kit from B-Bridged International (K1009–1, Santa Clara, CA, USA). The intra- and inter-assay coefficients of variation of esRAGE assay were 6.37 and 4.78–8.97%, respectively. We obtained cRAGE level by subtracting esRAGE from total sRAGE. The cRAGE/esRAGE ratio was than obtained. The GloMax^®^-Multi Microplate Multimode Reader was used for photometric measurements (Promega, Milan, Italy).

### 2.4. Glycated Albumin Quantification

Glycated albumin was measured by the enzymatic QuantILab^®^ Glycated Albumin assay (Instrumentation Laboratory, Milan, Italy) using the ILab650 system (Instrumentation Laboratory). The intra- and inter-assay coefficient of variations were 2.1 and 1.3% for GA and 1.2 and 1.0% for GA, respectively. 

### 2.5. AGE Quantification

AGE were quantified by measuring the fluorescence intensity of plasma samples at 414–445 nm after excitation at 365 nm, as previously reported [25,26], using a fluorescence spectrophotometer (The GloMax^®^, Promega). Fluorescence was expressed as the relative fluorescence intensity in arbitrary units (AU). AGE were then normalized for total protein content.

### 2.6. Statistical Analysis

Continuous variables were expressed as mean with standard deviation (SD). Categorical variables were summarized as percentages. Comparison between two groups was performed by Mann–Whitney test for continuous variables. Median follow-up was determined by the reverse Kaplan–Meier method. Univariate correlation was performed with Spearman’s correlation test. Kaplan–Meier estimates for survival curves to evaluate mortality in CKD patients with and without diabetes mellitus (DM). The Cox proportional hazards regression model was used to assess the association of variables with all-cause mortality in univariate and multivariable analyses. The effects of variables were adjusted for established and possible prognostic factors, such as age, malnutrition (measured as MIS) and dialysis. In multivariable modelling, the rule of thumb of 10 events per covariate was used. The proportional hazard assumption was evaluated by Schoenfeld residuals. Analysis was conducted using R software (R package version 3.5.2, R Core Team 2019, R Foundation for Statistical Computing, Vienna, Austria).

## 3. Results

Table 1 and Table 2 resume the main demographic and biochemical parameters of patients enrolled in the study, as well as their nutritional status. Mean eGFR was 25 (11) mL/min/1.73 m^2^. Based on eGFR, 17 patients were classified as G5, 60 as G4, 27 as G3b and 7 as G3a. According to body mass index (BMI), one patient was under weight, 30 were normal weight, 46 were over weight and 34 were obese. According to MIS, 69% of patients were malnurished. 

Mean levels of sRAGE, esRAGE and cRAGE were 2318.84 (1224) pg/mL, 649 (454) pg/mL and 1669 (901) pg/mL, respectively. The mean value of cRAGE/esRAGE ratio was 2.82 (0.96). AGE concentration was 3026 (766) arbitrary unit (AU), AGE/total protein content was 433 (109) AU/(g/dL), GA was 21.32 (7.45) % and MIS 6-0 (4.7).

**Figure 1:**
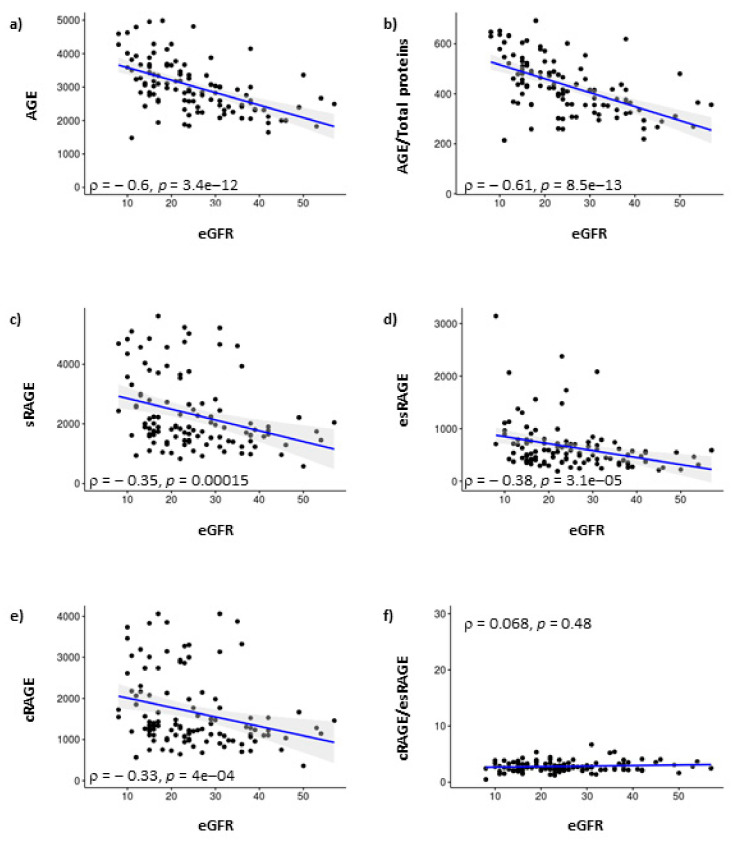
Correlations between eGFR (mL/min/1.73 m^2^) and AGE (arbitray unit) (panel **a**), AGE (arbitray unit)/Total proteins (g/dL) (panel **b**), sRAGE (pg/mL) (panel **c**), esRAGE (pg/mL) (panel **d**), cRAGE (pg/mL) (panel **e**) and cRAGE/esRAGE ratio (panel **f**). Data are visualized by scatter plots and corresponding regression lines (solid lines) demonstrating the correlations. For statistical evaluation, Spearman’s rank correlation analysis was used. Rho coefficients, *p*-value and CI 95% are shown.

We found a negative correlation of eGFR with AGE (*r* = −0.600; *p* < 0.001), AGE/total proteins (*r* = −0.613; *p* < 0.001), sRAGE (*r* = −0.352; *p* < 0.001), esRAGE (*r* = −0.384; *p* < 0.001) and cRAGE (*r* = −0.330; *p* < 0.001), but no evidence of association with cRAGE/esRAGE ratio (r = 0.068; *p* = 0.477) (Figure 1). Proteinuria, which is another marker of kidney damage, was not found to correlate with sRAGE and its forms (*p* > 0.05), but with AGE/total proteins (*r* = 0.206, *p* = 0.034). No significant associations have been found between AGE and markers of malnutrition (data not shown).

Sixty patients (54%) were affected by diabetes mellitus (DM). No evidence of difference was observed between non-DM and DM patients in the levels of AGE [3153 (763) AU vs. 2918 (757) AU], AGE/total proteins [451 (108) AU/(g/dL) vs. 418 (108) AU/(g/dL)], sRAGE [2517 (1309) pg/mL vs. 2148 (1130) pg/mL], esRAGE [708 (432) pg/mL vs. 598 (469) pg/mL], cRAGE [1809 (950) pg/mL vs. 1550 (847) pg/mL] and cRAGE/esRAGE ratio [2.70 (0.80) vs. 2.90 (1.04)] (*p* > 0.05 for all). GA% was higher in DM patients, instead [23.7 (8.6) % vs. 18.6 (4.6) %] (*p* < 0.001). 

During the follow-up, 28 patients started dialysis. Mean baseline levels of AGE [3529 (616) AU vs. 2857 (739) AU] (*p* < 0.0001), AGE/total proteins [517 (96) AU/(g/dL) vs. 405 (98) AU/(g/dL)] (*p* < 0.0001), sRAGE [2686 (1247) pg/mL vs. 2194 (1198) pg/mL] (*p* < 0.05) and esRAGE [823 (618) pg/mL vs. 590 (369) pg/mL] (*p* < 0.05) were significantly higher in this group. Mean levels of cRAGE were also higher [1863 (887) pg/mL vs. 1604 (902) pg/mL], but at a limit of statistical significance (*p* = 0.058). There was no evidence of difference in GA [20.4 (5.4) % vs. 21.6 (8.0) %] (*p* = 0.521) and cRAGE/esRAGE ratio [2.98 (0.82) vs. 2.75 (0.97)] (*p* = 0.122 for all) between the two groups. 

**Figure 2:**
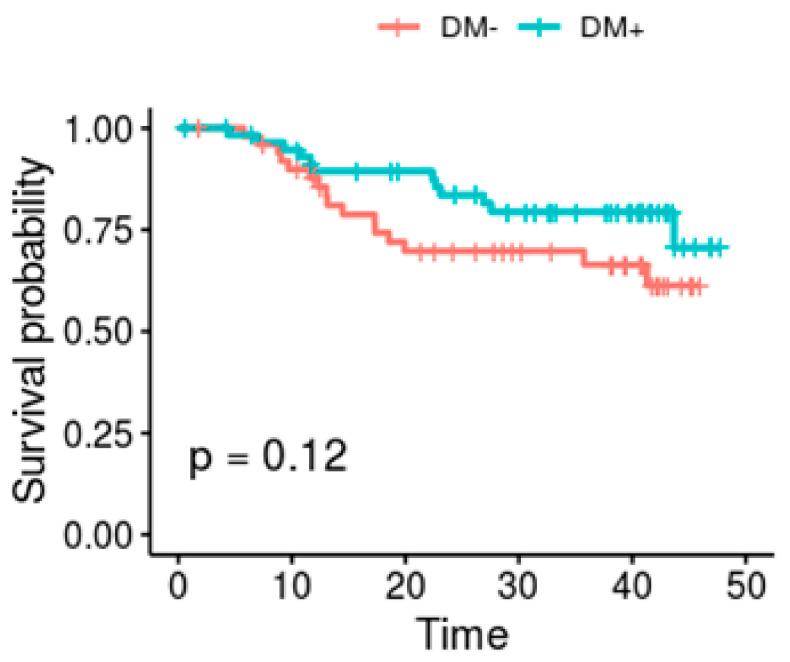
Kaplan–Meier survival analysis. Survival curves of CKD patients with (DM+, blue line) and without (DM−, red line) diabetes mellitus during the follow up time (months).

Twenty-eight patients died during the study. Among these patients, 16 were non-DM and 12 DM. Eight had started dialysis. There was no evidence of difference in mortality between DM and non-DM patients (*p* = 0.12) (Figure 2). 

Starting dialysis did not associate with an enhanced risk of all-cause mortality from univariate Cox proportional hazard regression (Table 3) and multivariate model (Supplementary Table S1). Differently, age (*p* < 0.001), AGE (*p* < 0.05), AGE/total proteins (*p* < 0.05), esRAGE (*p* < 0.01), cRAGE/esRAGE ratio (*p* < 0.05) and MIS (*p* < 0.01) did it (Table 3).

After adjusting for age and MIS, the associations were still significant for AGE (*p* < 0.05), esRAGE (*p* < 0.01) and cRAGE/esRAGE ratio (*p* < 0.05) (Table 4).

The association with AGE/total protein lost significance (*p* = 0.073). When eGFR was further introduced in the multivariate model as an additional adjusting factor, we still got statistically significance results for AGE (HR: 1.001, 95%CI: 1 to 1.001, *p* < 0.05), esRAGE (HR: 1.001, 95%CI: 1.0002 to 1.001, *p* < 0.01) and cRAGE/esRAGE ratio (HR: 0.585, 95%CI: 0.357 to 0.960, *p* < 0.01).

## 4. Discussion 

Over the last 27-year period, CKD has become one of the leading causes of death. Aging and the increasing prevalence of some traditional risk factors for CKD, such as DM and hypertension, contributed to more than half of the deaths from CKD in 2017 [27]. Currently, a reduction in risk factors and early identification of high-risk patients are the principal interventions devoted to reducing CKD mortality. The last one prompted us to evaluate the prognostic role on all-cause mortality of AGE and the different forms of sRAGE in a group of patients with advanced CKD. Previous studies, from our and other groups, have already explored the association of total sRAGE with the development and progression of CKD, CKD-related diseases, and CKD outcomes [15,17,28]. However, to our knowledge, this is the first study that evaluated the role of the different sRAGE forms as biomarkers for mortality in advanced CKD patients not yet on dialysis.

One of the main findings of our study was the inverse association between cRAGE/esRAGE ratio and the risk of mortality. Specifically, a difference of 0.1 unit in the ratio corresponded to a difference of about 6% in the risk of death. Additionally, an increase of 100 pg/mL of esRAGE and 100 AU of AGE were associated with an increased risk of mortality of about 10%.

AGE are molecules directly involved in the onset and progression of renal damage and are risk factors for CVD and mortality [13,29]. In non-CKD patients, hyperglycemia and oxidative stress are the main promoters of AGE production [30]. AGE are significantly higher in patients with a reduced renal function, even in the absence of hyperglycemia, with a non-significant difference between uremic patients with and without DM [11,31].

In accordance with these observations, we found an inverse association of AGE with eGFR and no differences between DM and non-DM patients. However, this observation is not entirely consistent with a previous study, which found higher AGE levels in end-stage renal disease (ESRD) patients with DM than non-DM [32]. Notably, our DM CKD patients had greater GA% than non-DM CKD, similarly to what we have previously observed in dialysis patients [17]. Considering that GA is a product of glycation that reflects early changes in glycemic control [33], the increased levels observed in DM should be considered a direct consequence of a worsened glycemic control and oxidative stress. Furthermore, GA seems a more reliable marker for glycemic control in CKD patients in which HbA1c suffers some limits, because it can both under- and over-estimate a patient’s glycaemia, depending on the disease state and drug therapy [22]. Differently from previous studies in dialysis patients [22], we found no evidence of association of GA with all-cause mortality, although not so far from statistical significance. Therefore, our data cannot definitively exclude that GA may have a prognostic role on mortality in CKD patients not yet on dialysis. Maybe, the number of patients enrolled in the study and the mortality rate were not enough to point out the potential prognostic role of GA. Although many studies have consistently demonstrated that reduced kidney filtration is the main determinant of AGE accumulation, the association of DM with AGE accumulation in the setting of uremia is still controversial. Nevertheless, our study agrees with previous studies demonstrating that AGE accumulation is a predictor of all-cause mortality in CKD [34,35]. Although AGE are mainly considered promoters of glomerular lesion, in vitro and pre-clinical studies suggested a potential role also in tubular injury [36,37,38,39], but no human studies have explored this issue yet. Therefore, additional studies exploring the associations between AGE and tubular injury markers could be helpful in identifying additional tools for disease monitoring.

In our patients, the single sRAGE forms and cRAGE/esRAGE ratio showed different prognostic roles on mortality. Before discussing this result, we must consider some premises. First, given that cRAGE and esRAGE are produced by different and independent mechanisms [20,40], their ratio reflects if a disease and/or an intervention can proportionally or disproportionally affect the two forms. Therefore, their reciprocal change might associate to patient’s outcome differently from each single form, just as we observed in our patients. Moreover, the ratio lost the negative association with eGFR, thus resulting independent of kidney filtration. 

Reduced renal function is indicated as the main determinant of sRAGE accumulation [16,41,42]. sRAGE is a decoy receptor that prevents activation of membrane-bound RAGE and RAGE-related detrimental effects. A reduction in sRAGE levels has been observed in many diseases (i.e., hypertension, coronary artery diseases, chronic obstructive pulmonary disease, hyperthyroidism, and rheumatic arthritis and Alzheimer disease), and it has been associated to their onset and progression [18,43,44]. Increased sRAGE levels have been observed, instead, in DM and CKD, and they associate with a worsened patient outcome [16,42,45,46]. Therefore, sRAGE cannot be considered a universal biomarker, because its levels strongly depend on the type of disease.

DM affected half of the patients in our study. Although DM promotes AGE and sRAGE accumulation, DM and non-DM patients did not differ either in sRAGE, nor in cRAGE and esRAGE levels. These data confirm previous observation that DM and glycemic control have no influence on sRAGE accumulation in CKD, because their levels are mainly due to the decreased removal by the kidney [11,47]. However, these studies just focused on total sRAGE, not on the different isoforms and their ratio. Being independent of eGFR, to our opinion cRAGE/esRAGE ratio could represent an interesting marker in risk stratification, because it might reflect the activation/modulation of other mechanisms that can differently affect the different sRAGE forms, regardless of renal function. Usually, RAGE expression on cell membrane is low. AGE and other molecules can bind to RAGE and activate a transcriptional process that increases RAGE expression, promotes the inflammatory response, downregulates the synthesis of esRAGE form and increases the production of cRAGE by shedding the membrane-bound receptor [19,20,21]. cRAGE levels can thus reflect the increased activation of the AGE/RAGE axis and an existing pro-inflammatory status. However, RAGE shedding is also a protective mechanism to block AGE and RAGE activation. Lee et al. [48] observed a strong association of ADAM10 (a disintegrin and metalloproteinase 10) activity with cRAGE, but not esRAGE, thus confirming that different mechanisms are involved in producing the two forms. Therefore, this proteolytic activity seems the main determinant of the reduction in the risk of death as the ratio increases. In our study, we did not quantify specific molecules, such as ADAM10 [20], involved in RAGE shedding. However, we can hypothesize that patients displaying high levels of this enzyme might have a better prognosis. Therefore, this molecule could be an interesting therapeutic target that could help to improve the circulating levels of cRAGE and its protective role. 

Our study has some limits. We included patients at different stages of CKD, which have been experiencing the disease for a different time that could not be quantified. In fact, many patients had already an advance stage of CKD and an incomplete clinical history when they came to our outpatient clinic for the first time. Furthermore, the study was performed in a relatively small group of patients. However, the application of stringent exclusion criteria let us to evaluate a quite homogeneous group of individuals that are representative of most CKD patients attending nephrology outpatient clinics. Although the number of subjects was relatively small, they were observed for a sufficient time to describe the impact of mortality in a cohort of seniors CKD patients, and the number of events was consistent with the overall purpose of the study. Starting dialysis is a potential additional confounding factor, because it can independently increase the mortality rate [49,50]. There was no evidence of association of starting dialysis and mortality in our patients during the observation time, and when we included it as an additional adjusting factor in the multivariable Cox proportional hazard regression model, esRAGE and the ratio were still significantly associated with all-cause mortality. Maybe just longer observation time could give additional information whether AGE, sRAGE forms and dialysis are competing risk factors for all-cause mortality. A final consideration regarding DM status; in fact, it would be interesting to evaluate DM as an interaction with AGE rather than revealing the main effects of DM. However, a larger study is necessary to have enough power to evaluate the possible presence of interaction effects.

## 5. Conclusions

AGE and sRAGE are molecules strongly related to CKD. In particular, esRAGE and cRAGE/esRAGE ratio seem to be useful and supportive tools to identify high-risk patients. Future studies focused on the mechanisms that can modulate cRAGE production can also help the identification of both additional biomarkers and potential therapeutic targets.

## Figures and Tables

**Table 1:** Cohort characteristics.

	Overall Cohort
N	111
Age, years	78 (10)
Males, %	71
Diabetes, %	54
Previous cardiovascular events, %	16
Malnourished at MIS, %	69
eGFR, mL/min/1.73 m^2^	25 (11)
Fasting blood glucose, mg/dL	116 (39)
Uric acid, mg/dL	6.2 (1.5)
Hemoglobin, gr/dL	12.4 (1.5)
Proteinuria, mg/24 h	1070 (1464)

Data are expressed as number or percentage or mean (standard deviation). eGFR, estimated glomerular filtration rate; MIS, malnutrition inflammatory score; N, number.

**Table 2:** Nutritional status.

	Overall Cohort
MIS	6.0 (4.7)
Albumin, gr/dL	4.1 (0.4)
Prealbumin, mg/dL	28.7 (5.7)
Total cholesterol, mg/dL	168 (37)
Transferrin, mg/dL	230 (40)
CRP, mg/dL	0.46 (0.76)
nPCR, mg/kg/24 h	759 (239)
BMI, kg/m^2^	27.9 (4.9)

BMI, body mass index; CRP, C reactive protein; MIS, malnutrition inflammation score; nPCR, normalized protein catabolic rate.

**Table 3:** Associations with all-cause mortality at univariate Cox proportional hazard regression. Hazard ratios for AGE, cRAGE, esRAGE, sRAGE and are reported for 100-unit increments.

Variable	Hazard Ratio	95% CI	*p*
Age	1.100	1.041 to 1.163	**<0.001**
AGE	1.051	1.002 to 1.103	**0.04**
AGE/total proteins	1.004	1.000 to 1.007	**0.03**
esRAGE	1.083	1.027 to 1.141	**0.003**
cRAGE/esRAGE	0.539	0.331 to 0.878	**0.013**
MIS	1.079	1.025 to 1.137	**0.004**
sRAGE	1.025	0.997 to 1.055	0.084
cRAGE	1.018	0.979 to 1.059	0.373
GA%	0.971	0.909 to 1.037	0.381
eGFR	0.969	0.932 to 1.008	0.123
Dialysis	1.745	0.715 to 4.260	0.222

AGE, advanced glycation end products; cRAGE, cleaved receptor for advanced glycation end products; eGFR, estimated glomerular filtration rate; esRAGE, endogenous secretory receptor for advanced glycation end products; GA, glycated albumin; MIS, malnutrition inflammatory score; sRAGE, soluble receptor for advanced glycation end products. *p* values less than 0.05 are indicated in bold.

**Table 4:** Association with all-cause mortality at multivariable Cox proportional hazard regression. Hazard ratios for AGE, cRAGE, esRAGE and sRAGE are reported as 100-unit increments.

Variable	Hazard Ratio	95% CI	*p*
esRAGE	1.087	1.023 to 1.156	**0.007**
cRAGE/esRAGE	0.572	0.347 to 0.943	**0.029**
AGE	1.062	1.005 to 1.121	**0.031**
AGE/total proteins	1.004	1.000 to 1.007	0.073
sRAGE	1.020	0.987 to 1.054	0.244
cRAGE	1.007	0.965 to 1.052	0.745
GA%	0.924	0.849 to 1.006	0.068

The hazard ratios are reported resulting from a multivariable model adjusted for age and malnutrition inflammatory score (MIS). AGEs, advanced glycation end products; cRAGE, cleaved receptor for advanced glycation end products; esRAGE, endogenous secretory receptor for advanced glycation end products; GA, glycated albumin. *p* values less than 0.05 are indicated in bold.

## Supplementary Materials

The following are available online at https://www.mdpi.com/2227-9059/8/12/638/s1. Supplementary Table S1. Association with all-cause mortality at multivariable Cox proportional hazard regression adjusted for age, MIS (malnutrition inflammatory score) and dialysis status.
