# Supplementary material for: Advanced Glycation End Products (AGE) and Soluble Forms of AGE Receptor: Emerging Role as Mortality Risk Factors in CKD
Source: Biomedicines. 2020 Dec 21;8(12):638. doi: 10.3390/biomedicines8120638 (PMC7767383; doi:10.3390/biomedicines8120638)
Supplement: Supplementary file 1 [file biomedicines-08-00638-s001.pdf]

Supplementary Table

Table S1. Association with all-cause mortality at multivariable Cox proportional hazard regression adjusteed for age, MIS (malnutrition inflammatory score) and dialysis status.

	Variable		Age		MIS		Dialysis	
	**HR**	***p***	**HR**	***p***	**HR**	***p***	**HR**	***p***
**esRAGE**	1.001	0.044	1.102	0.006	1.039	0.244	1.795	0.285
**cRAGE/esRAGE**	0.621	0.052	1.096	0.01	1.055	0.083	2.011	0.168
**AGEs**	1	0.11	1.09	0.011	1.065	0.049	1.651	0.351
**AGEs/ Total proteins**	1.002	0.289	1.085	0.015	1.062	0.065	1.72	0.353
**sRAGE**	1	0.372	1.093	0.008	1.046	0.205	2.235	0.11
**cRAGE**	1	0.863	1.091	0.009	1.059	0.097	2.403	0.076
**GA**	0.925	0.074	1.101	0.008	1.089	0.019	0.925	0.085

The hazard ratios are reported resulting from a multivariable model adjusted for age, MIS (malnutrition inflammatory score) and dialysis status. AGEs, advanced glycation end products; cRAGE, cleaved receptor for advanced glycation end products; esRAGE, endogenous secretory receptor for advanced glycation end products; GA, glycated albumin.
